# Comparing Verum and Sham Acupuncture in Fibromyalgia Syndrome: A Systematic Review and Meta-Analysis

**DOI:** 10.1155/2019/8757685

**Published:** 2019-08-25

**Authors:** Jiwon Kim, Su-Ryun Kim, Hyangsook Lee, Dong-Hyun Nam

**Affiliations:** ^1^Department of Biofunctional Medicine and Diagnosis, College of Korean Medicine, Sangji University, Wonju, Republic of Korea; ^2^Department of Anatomy, College of Korean Medicine, Kyung Hee University, Seoul, Republic of Korea

## Abstract

**Objectives:**

Acupuncture is often used for relieving symptoms of fibromyalgia syndrome (FMS). Our aim is to ascertain whether verum acupuncture is more effective than sham acupuncture in FMS.

**Methods:**

We collected RCTs to investigate the effects of verum acupuncture and sham acupuncture on pain, sleep quality, fatigue, and general status in FMS patients. The databases used for data retrieval were PubMed, Central Cochrane, EMBASE, PsycINFO, CNKI, VIP, OASIS, KoreaMed, and RISS. Selection/exclusion from the retrieved records was performed according to prespecified criteria, and the final selected records were assessed according to the Cochrane risk of bias tool. The results of the included trials were synthesized on the basis of outcomes, and subgroup analysis depended on the type of add-on sham acupuncture that was performed.

**Results:**

Ten RCTs (690 participants) were eligible, and eight RCTs were eventually included in the meta-analysis. The synthesis showed a sizable effect of verum acupuncture compared with sham acupuncture on pain relief (standardized mean difference (SMD) −0.49, *Z* = 3.26, *P*=0.001; *I*^2^ = 59%), improving sleep quality (SMD −0.46, *Z* = 3.24, *P*=0.001; *I*^2^ = 0%), and reforming general status (SMD −0.69, *Z* = 6.27, *P* < 0.00001; *I*^2^ = 4%). However, efficacy on fatigue was insignificant (SMD −0.10, *Z* = 0.51, *P*=0.61; *I*^2^ = 46%). When compared with a combination of simulation and improper location of needling, the effect of verum acupuncture for pain relief was the most obvious.

**Conclusions:**

Verum acupuncture is more effective than sham acupuncture for pain relief, improving sleep quality, and reforming general status in FMS posttreatment. However, evidence that it reduces fatigue was not found.

## 1. Introduction

Fibromyalgia syndrome (FMS) is a disorder with medically unexplained symptoms and characterized by chronic, widespread pain and multisite muscular tenderness for more than 3 months [1]. Patients with FMS suffer not only from pain but also from a range of symptoms including cognitive dysfunction, fatigue, sleep disturbance, and somatic and psychological distress [2–5]. Because there is currently no cure for it, management of FMS usually focuses on symptom relief [6]. In this context, a number of patients with FMS have increasingly taken interest in complementary and alternative medicine and have used one or more complementary and alternative therapies [7]. Acupuncture, in particular, has steadily gained popularity in the German population: a German consumer survey involving patients with FMS reported that acupuncture accounted for 28.5% of complementary and alternative medicine usage [7] and another survey reports that 15–20% of FMS patients seek acupuncture treatment or trigger point injections [8].

While acupuncture is one of the widely supported nonpharmacological treatment options for symptom management for various musculoskeletal disorders including chronic pain [9], low back pain [10], and knee osteoarthritis [11], the Association of the Scientific Medical Societies in Germany recommended acupuncture should be avoided in FMS patients [3]. This recommendation basically stems from the inconsistent findings that acupuncture is more effective than sham acupuncture in FMS [12].

Sham acupuncture refers to a placebo control used in clinical trials of acupuncture [13]. Sham acupuncture has been used to reveal several, specific or nonspecific, essential components related to acupuncture treatment [14]. Some researchers have used devices like a stage dagger as a sham needle, which may exert an effect similar to acupressure [15, 16]. Others even pretended needling [17] or used superficial needles at nonacupoints [18, 19] or inappropriate acupoints according to traditional acupuncture theories [20, 21]. Thus far, sham acupuncture is criticized for not being physiologically inert [22, 23], and its efficacy may vary depending on the disease or the method applied [24].

Although several previous systematic reviews supported pain reduction by acupuncture in FMS patients [25–27], others refuted the efficacy of acupuncture for pain reduction when compared with sham acupuncture, suggesting that acupuncture might have a mere placebo effect in patients with FMS [28, 29]. The conclusions of those meta-analyses were derived from the trials conducted prior to 2010. On the other hand, many recently conducted clinical trials reported that verum acupuncture is more effective in alleviating the symptoms of FMS patients than sham acupuncture [30–33]. Such contradicting effects and the existence of various sham acupunctures make it difficult to draw a firm conclusion concerning the use of acupuncture in FMS patients. Therefore, this systematic review aimed to draw a conclusion on whether verum acupuncture is more effective in managing FMS symptoms than sham acupuncture and to investigate whether there is a difference in the effect size among the various types of the sham acupuncture.

## 2. Methods

### 2.1. Protocol and Registration

The protocol registration number of this study is PROSPERO 2018:CRD42018076279 and is accessible from https://www.crd.york.ac.uk/prospero/display_record.php?RecordID=76279.

### 2.2. Eligibility Criteria

#### 2.2.1. Types of Study

This is a randomized controlled trials (RCTs).

#### 2.2.2. Patients

Patients over the age of 17 years were diagnosed with FMS according to the American College of Rheumatology (ACR) criteria.

#### 2.2.3. Interventions

Verum acupuncture was defined in this study as needling acupoints according to a traditional meridian theory or neurophysiology and anatomy, i.e., western medical acupuncture [34]. It was limited to acupuncture in which skin is pierced using a thin, pointed needle, e.g., manual acupuncture, electroacupuncture, and/or auricular acupuncture, and studies testing acupuncture combined with other treatments were also included if the same cointerventions were given to the control group. Dry needling using a syringe, transcutaneous electrical nerve stimulation (TENS), laser acupuncture, embedded acupuncture, pharmacopuncture, and acupressure were excluded.

#### 2.2.4. Control Groups

We only sought studies that adopted sham acupuncture in the control group. Sham acupuncture included simulating acupuncture, sham needle device, needling to acupoints not related to FM or nonacupoints, and a combination of those sham acupuncture methods.

#### 2.2.5. Outcomes

In order to evaluate the short-term effects, we selected studies in which the intensity of pain and fatigue, sleep quality, and general status were assessed at baseline and during the final intervention. The primary outcome was the intensity of pain, which can be measured by various scales (e.g., visual analog scale (VAS), pain scale in the fibromyalgia impact questionnaire (FIQ), McGill pain questionnaire (MPQ), regional pain score (RPS), numerical rating scale (NRS), and multidimensional pain inventory (MPI)). The secondary outcomes were severity of fatigue, sleep quality, and general status in FMS patients, which were measured by various tools such as VAS, multidimensional fatigue, Pittsburgh sleep quality index (PSQI), and FIQ. Clinical trial reports that did not provide outcomes that changed in value, brought about by the interventions, were excluded from the meta-analysis.

### 2.3. Search and Study Selection

This review included trials published from 1990 to August 2018 without language restriction and limitation of publication type, e.g., original article, thesis, and/or conference proceedings. A total of 9 databases were searched: PubMed; Cochrane Central Register of Controlled Trials; EMBASE; PsycINFO; two Chinese databases, namely, CNKI (China National Knowledge Infrastructure) and Chongqing VIP; and three Korean databases, namely, OASIS (Oriental medicine Advanced Searching Integrated System), KoreaMed, and RISS (Research Information Sharing Service). The keywords used for the search were as follows: (“fibromyalgia” [MESH] OR fibromyositis OR “fibromyalgia-fibromyositis syndrome” OR “myofascial pain syndrome”) and (acupuncture OR auriculotherapy OR electroacupuncture OR acupoint OR needling).

The specific keywords used to search each database are presented in the Appendix ([Supplementary-material supplementary-material-1]).

At first, we eliminated any duplicate studies by checking the title, journal, and publication year, and then the articles were reviewed and selected based on the eligibility criteria by two independent reviewers (Kim J. W. and Kim S. R.). If there were disagreements between the reviewers, they reached an agreement through a discussion. When a consensus was still not reached, another reviewer (Nam D. H.) participated in the discussion and a final decision was made. With regard to missing data, we contacted the authors of the articles in order to acquire detailed information. If we could not acquire the data, we excluded the trial from the meta-analysis and summarized it comprehensively.

### 2.4. Assessment of Risk of Bias (ROB)

The ROB of the included trials was assessed by the Cochrane ROB assessment tool [35]: random sequence generation, allocation concealment, blinding of participants and personnel, blinding of outcome assessment, incomplete outcome data, selective reporting, and other bias. As blinding of the acupuncture practitioner is virtually impossible due to the nature of the intervention, blinding of the practitioner was not formally included in the ROB assessment. Instead, we looked for any mentioning of practitioner blinding or related issues in the included reports to descriptively evaluate any impact that a lack of practitioner blinding might have on the trial results.

### 2.5. Adverse Events

To investigate the potential risks of verum and sham acupuncture in FMS, we summarized the adverse events reported in selected studies.

### 2.6. Data Analysis and Synthesis

The data were synthesized using the Review Manager software program (version 5.3, Nordic Cochrane Centre, The Cochrane Collaboration, Copenhagen, Denmark). To investigate the difference in the effect size between verum acupuncture and various types of sham acupuncture, sham acupuncture was classified into three categories: simulated acupuncture on appropriate points (SA-AP), acupuncture on inappropriate points (A-IP), and simulated acupuncture on inappropriate points (SA-IP). SA-AP does not involve the piercing of the skin on acupoints related to FMS, but instead, a bandage, guide tube, Park's sham, or power-off electronic equipment is used to create a placebo effect. A-IP involves piercing nonacupoints or points located some distance away from the actual acupoints related to FMS. Finally, SA-IP involves neither piercing the skin on nonacupoint nor acupoints unrelated to FMS.

For continuous outcomes, the mean difference (MD) and 95% CI were calculated. If different scales were used to measure the same parameter (e.g., intensity of pain), we calculated the standardized mean difference (SMD). We used a random-effects model with an inverse variance method as the clinical characteristics of the included studies were expected to highly vary. In case of a multiarm RCT, the number of participants in the verum acupuncture group was divided by the number of the sham acupuncture groups in order to avoid overweighting the verum acupuncture group in the meta-analysis.

Heterogeneity among the studies was evaluated by a chi-squared test with a *P* value of less than 0.1. To evaluate inconsistencies among the included studies, the *I*^2^ statistic was used. When the value of the *I*^2^ statistic was over 50%, we considered that to mean there was substantial heterogeneity among included studies [36]. The small study effect, i.e., a tendency for estimates of the intervention to be more beneficial in smaller trials, was also evaluated with funnel plots when more than 10 studies were included in the analysis.

### 2.7. Assessment of Evidence Quality

Evaluation of the evidence level was performed according to the Grading of Recommendations Assessment, Development, and Evaluation (GRADE) guideline development tool [37]. The quality of evidence was assessed in terms of study design, risk of bias, inconsistency, indirectness, imprecision, and other considerations. Because the GRADE does not provide concrete, detailed criteria for the quality of evidence, we assessed it by applying a guidance which National Evidence-based Healthcare Collaborating Agency (NECA) suggested [38].

## 3. Results

### 3.1. Study Selection

**Figure 1:**
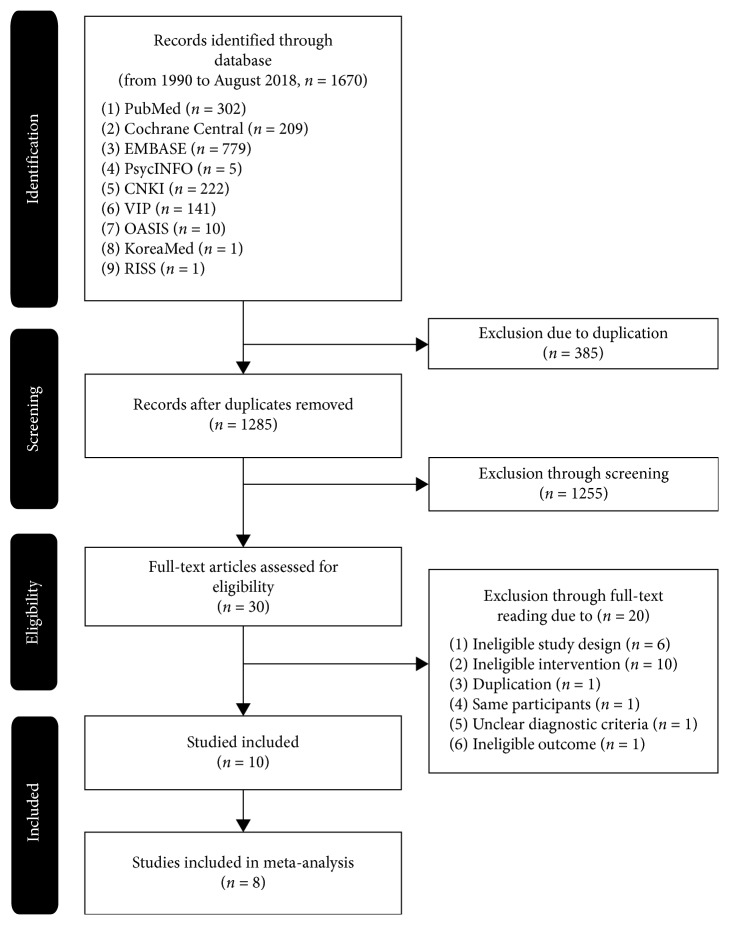
Flow diagram of the selection process.

We identified 1670 records through searching multiple databases, and 385 of them were excluded as they were duplicates. We excluded 1255 records through screening the titles and abstracts. After full-text reading, 20 records were excluded for the following reasons: ineligible study design (6 studies), ineligible intervention for treatment group or control group (10 studies), duplicated study (1 study), the same participants (1 study), unclear diagnostic criteria (1 study), and discussing factors other than pain, quality of sleep, fatigue, and general status (1 study). Ten RCTs (*n* = 690) met the inclusion criteria, and two RCTs [12, 39] were excluded from our meta-analysis as all of the results were not reported. Data for synthesis, e.g., mean, standard deviation, *t*-score, and/or *P*-value, were not reported in the two RCTs [12, 39]. We tried to contact the authors via e-mail to acquire the missing data, but only one author replied who said that they no longer had access to the raw data. Finally, participants of eight RCTs (*n* = 561) were included in the meta-analysis. The PRISMA chart in this systematic review is shown in Figure 1.

The ACR 1990 diagnostic criteria [40] were used in all included RCTs. In most of the included RCTs, the female proportion was considerably higher than the male and four RCTs consisted only of females [30, 33, 41]. In one RCT [42], there were more male participants than female participants, and in another RCT, the gender ratio was not reported [39].

The duration of acupuncture treatment varied from 1 session to over 12 weeks' worth of sessions, with a frequency of 1–3 sessions per week. Manual acupuncture was used as treatment intervention in eight RCTs [12, 21, 30–33, 39, 41], and electroacupuncture was used in two RCTs [42, 43]. In seven RCTs [12, 21, 31–33, 41, 43], the acupoints were at fixed locations in both the verum and sham acupuncture groups, whereas in three RCTs [30, 39, 42] the acupoints were combined with other points according to the patient's pattern or symptoms.

Two 3-arm RCTs [32, 39], two 4-arm RCTs [12, 21], and six RCTs with one control group [30, 31, 33, 41–43] were included. Out of the eight RCTs in the meta-analysis, four RCTs [21, 32, 33, 43] involved the application of simulated acupuncture at the same points targeted in verum acupuncture in a control arm, and a nonacupoint acupuncture was set in a control arm in four RCTs [21, 31, 32, 42]. Simulation at nonacupoints as a control arm was performed in two RCTs [21, 41], and simulation at different acupoints from the verum acupuncture in the control group was organized for one RCT [30]. Those sham acupunctures were categorized into the SA-IP category [21, 30, 41]. All of the included studies are summarized in Table 1.

### 3.2. Risk of Bias in the Included Studies

**Figure 2:**
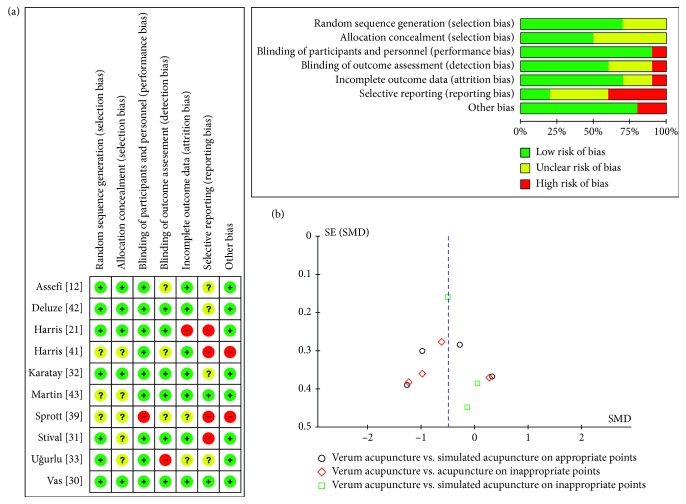
(a) Risk of bias and (b) funnel plot of pain.

The results of evaluating the risk of bias are presented in Figure 2(a). In a random sequence generation, seven of ten RCTs [12, 21, 30–33, 42] were assessed as low risk, and the others were unclear according to the randomization method. Only five RCTs [12, 21, 30, 32, 42] were considered as low risk in allocation concealment. Participant blinding was appropriate in all but one RCT [39]; interaction such as conversation between the participants and the practitioner was not adequately limited. Regarding the practitioner blinding which was not included in the ROB assessment, there was no mentioning or assessment of practitioner blinding in the included trial reports. Blinding of the outcome assessor in only six RCTs [21, 30–32, 42, 43] was obvious. A high dropout rate in one RCT [21] caused it to be assessed as high risk for incomplete outcome data. In selective reporting, only two RCTs [30, 43] were considered as low risk due to the absence of their published protocols or lack of outcome tools generally used in FMS. In other sources, two RCTs [39, 41] were assessed as high risk. In one RCT [41], the authors declared that they did not analyze the effects of verum acupuncture versus sham acupuncture, and pain outcome was only used as a covariate. In the other RCT [39], the validity of the no difference in baseline outcomes among groups was inadequate.

### 3.3. Primary Outcome (Intensity of Pain)

**Figure 3:**
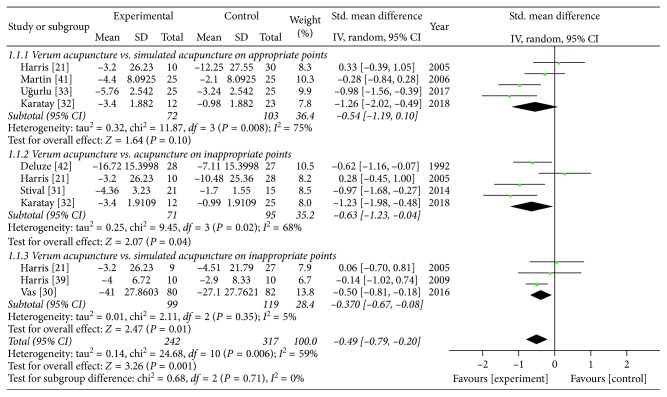
Comparison of the verum acupuncture and sham acupuncture groups in terms of pain.

From having synthesized eight RCTs [21, 30–33, 41–43], we confirmed the significant pain reduction effect of verum acupuncture compared with sham acupuncture (SMD −0.49 (95% CI: −0.79, −0.20), *Z* = 3.26, *P*=0.001), and its heterogeneity was substantial (*I*^2^ = 59%). When the analyses were restricted to the RCTs comparing verum acupuncture with SA-AP [32, 33, 41, 43] and A-IP [31, 32, 41, 42], separately, the beneficial pain relief effect remained (SA-AP: SMD −0.54 (95% CI: −0.19, 0.10), *Z* = 1.64, *P*=0.10; A-IP: SMD −0.63 (95% CI: −1.23, −0.04), *Z* = 2.07, *P*=0.04), but the heterogeneities were all substantial (SA-AP: *I*^2^ = 75%; A-IP: *I*^2^ = 68%). In the SA-IP [21, 30, 41], the effect size was still significant (SMD −0.37 (95% CI: −0.67, −0.08), *Z* = 2.47, *P*=0.01) and its heterogeneity was of no importance (*I*^2^ = 5%). The heterogeneity among subgroups classified by the types of sham acupuncture was of no importance (*I*^2^ = 0%). A forest plot of the pain reduction effect is shown in Figure 3, and its small publication bias can be identified through symmetry as shown in Figure 2(b).

Two RCTs excluded from the meta-analysis [12, 39] showed that verum acupuncture alleviated pain, but its effect on pain relief was not significant in comparison with sham acupuncture because of the similar effects noted in sham acupuncture.

### 3.4. Secondary Outcomes

#### 3.4.1. Sleep Quality

Synthesis of three RCTs [32, 42, 43] showed that verum acupuncture over sham acupuncture improved sleep quality significantly (SMD −0.46 (95% CI: −0.75, −0.18), *Z* = 3.24, *P*=0.001) and that the level of its heterogeneity was very low (*I*^2^ = 0%). One 4-arm RCT [12] from the meta-analysis reported that no significant differences were found between the verum acupuncture and the pooled sham acupuncture for sleep quality, though verum acupuncture rapidly improved sleep quality.

#### 3.4.2. Fatigue

Three RCTs [21, 33, 43] were included in the meta-analysis and showed that there was no evidence for the superiority of verum acupuncture over SA-AP for improving fatigue (SMD −0.10 (95% CI: −0.51, 0.30), *Z* = 0.51, *P*=0.61; heterogeneity *I*^2^ = 46%). One RCT [12] not included in the meta-analysis also demonstrated that both verum and sham acupuncture improved fatigue, but there was no significant difference between the verum and the sham acupuncture.

#### 3.4.3. General Status

**Figure 4:**
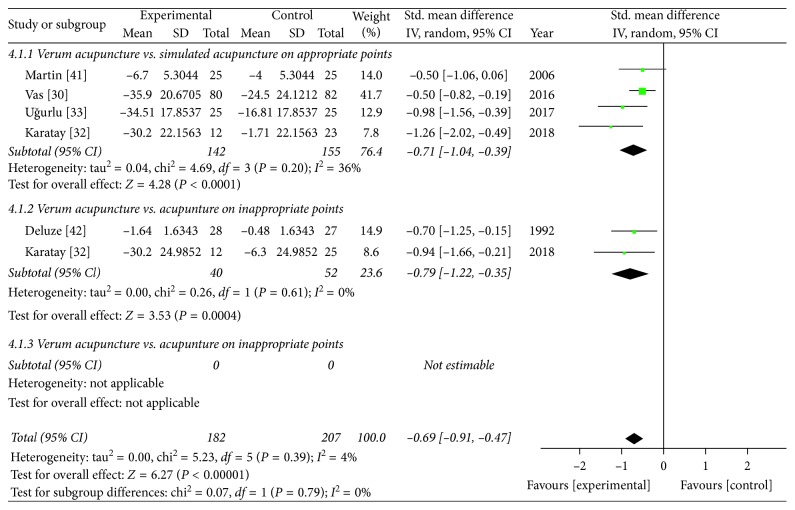
Comparison of the verum acupuncture and sham acupuncture groups in terms of general status.

A total of five RCTs [30, 32, 33, 42, 43] in the meta-analysis showed that the positive effect of verum acupuncture on improvement of general status was more significant than that of sham acupuncture (SMD −0.69 (95% CI: −0.91, −0.47), *Z* = 6.27, *P* < 0.00001), and its heterogeneity might not be important (*I*^2^ = 4%). A forest plot of general status is presented in Figure 4, and its funnel plot is omitted because the number of the included studies is too small. In one RCT eliminated from the meta-analysis [39], general status of patients intended to improve immediately after verum acupuncture, but it did not in both SA-AP and waitlist group.

### 3.5. Evidence Quality

The results of GRADE assessments are shown in Table 2. The evidence level of verum acupuncture's effect over sham acupuncture on pain relief was assessed as moderate. It was downgraded as very low in the SA-AP and as low in the A-IP; meanwhile, it was assessed as high in the SA-IP. In fatigue and sleep quality, the evidence level was low and high, respectively. The evidence levels of verum acupuncture's effect over sham acupuncture in general status ranged from moderate to high.

### 3.6. Adverse Events

Five RCTs [12, 30, 32, 42, 43] reported occurrences of adverse events. Adverse events included local edema, mild bruising, soreness at the sites of needle insertion, worsening symptoms, tiredness, headache, and mild vasovagal symptoms. A case of pulmonary embolism occurred in the SA-AP, but a direct association between the pulmonary embolism and the simulated electroacupuncture was rejected [43]. In two RCTs [12, 43], although the incidence was lower in the simulated acupuncture group than in the verum acupuncture group, mild adverse events such as local discomfort, mild bruising, soreness, and tiredness were still considerable in the simulated acupuncture group.

## 4. Discussion

Through this systematic review with meta-analysis, we found that verum acupuncture is more effective than sham acupuncture in the relief of pain, improvement of sleep quality, and recovery of general statuses in FMS posttreatment. The effect size on pain relief was not large, and the quality of evidence was moderate. It implies that verum acupuncture seems to be more effective in pain relief than sham acupuncture, but the true effect can be substantially different. In the SA-IP type, the most obvious sham acupuncture, the effect on pain relief was the most significant and its evidence quality was high. When verum acupuncture was compared with the SA-AP type, which can have similar effects to acupressure, the effect was insignificant and its evidence level was downgraded to very low.

No evidence that verum acupuncture reduces fatigue more effectively than sham could be found. However, its effectiveness on improvement of sleep quality and general status was clear, and the level of evidence was high. Although transient adverse events such as local pain, tiredness, and mild vasovagal symptoms had been reported, serious adverse events had not been associated with acupuncture in FMS.

### 4.1. Interpretations

Muscle pain and tenderness are the most common symptoms in FMS patients, and achieving pain relief is an essential factor in the management of FMS patients. Despite the significant analgesic effect of verum acupuncture versus sham acupuncture, the high heterogeneity downgraded the level of evidence. The substantial heterogeneity could have been due to the diversity in pain sensitivity, frequency and duration of the intervention, selection of needling points, and stimulus intensity. Etiopathogenetic hypotheses regarding FMS have suggested the possibility of varying degrees of peripheral nociception and central sensitization [44, 45]; some researchers reported that there were various responses to acupuncture treatment depending on the FMS patients' sensitivity to pain [46]. Thus, the levels of pain sensitivity of the participants in the included RCTs might also vary. Furthermore, the diagnostic criteria for FMS, which has been frequently changed, may also have increased the heterogeneity among the trials. The ACR 1990 diagnostic criteria focused on the number of tenderness points, but by virtue of the ACR 2011 diagnostic criteria, accompanied symptoms such as sleep disturbances, fatigue, poor cognition, headache, and depression also became important factors [4]. Considering that there is a high likelihood that subjective perspectives play a role when applying the FMS diagnostic criteria, even if all of the participants in this systematic review were recruited according to the ACR 1990 diagnostic criteria, changes in the FMS diagnostic criteria may have affected the recruiting process of FMS patients. In our post hoc analysis conducted chronologically, we identified the prominent differences in effect size and heterogeneity between the results of the RCTs conducted before 2011 (SMD −0.12 (95% CI: −0.43, 0.19), *Z* = 0.77, *P*=0.44; heterogeneity *I*^2^ = 21%) and the RCTs conducted after 2011 (SMD −0.88 (95% CI: −1.22, −0.54), *Z* = 5.11, *P* < 0.00001; heterogeneity *I*^2^ = 41%). Diagnostic criteria for FM are constantly being revised to improve sensitivity and specificity [47].

Regarding the intensity of stimulation, we could not have confidence that the stimulation intensity of the manual acupuncture was well controlled. Only four studies [21, 30, 33, 41] reported whether or not they performed needle manipulation in relation to stimulus intensity to elicit “De Qi” or needle sensation. In two of the RCTs [30, 41], the practitioner manipulated the needles to achieve “De Qi” sensation, but not in the other two [21, 33].

Effects of acupuncture on widespread muscular pain such as FMS may be explained through complex neurohumoral factors in the brain, spinal cord, and peripheral tissues; it involves modulation of endogenous opioids [48–52], release of serotonin [32], and reducing substance *P* levels [53]. A study using a brain positron emission tomography technique showed that *μ*-opioid receptor-binding ability was stronger in verum acupuncture than in sham acupuncture even though there was no significant difference in clinical pain relief in both groups [41, 54]. Simulated acupuncture can also give stimuli similar to verum acupuncture, but the strength of the stimulus is relatively weak. Recently, findings that dry needling has the ability to reduce the release of substance *P*, in a dose-dependent manner, and elevate opioid levels in rabbits have been reported [50, 53]. However, there is still a lack of well-designed clinical trials that directly demonstrate the dose-dependent effects of acupressure.

Although fatigue is one of the main symptoms in FMS, it has not been recognized as important as the pain [55]. Chronic fatigue syndrome (CFS) is very similar to FMS, especially according to the ACR 2011 diagnostic criteria, and is also one of the common concomitant diseases of FMS [56]. Recent systematic reviews of the effects of acupuncture on CFS concluded that acupuncture is effective in managing fatigue-related symptoms in CFS [57, 58]. Differences in the acupoints usually used in FMS and CFS may have led to differences in efficacy regarding fatigue improvement. The transport points on the back are mainly used for managing fatigue in CFS, whereas the acupoints on the paravertebral area are often neglected in FMS. In this meta-analysis, the effects of verum acupuncture versus simulated acupuncture in an RCT [21] not using the acupoint of the bladder meridian along the spine was unfavorable, whereas the effects in the two RCTs with the acupoints on the back were beneficial. However, this should be interpreted in this manner with caution as the recent systematic review of CFS [57] drew conclusions based on studies with a high risk of bias.

Our meta-analysis shows that verum acupuncture over sham acupuncture has improved general status in FMS. FMS is understood as a disorder of the pain signal process in the central nervous system and is accompanied by various symptoms. FIQ is a representative tool to measure the main components of the general health status that is affected by FMS [59], and four out of five RCTs that have reported changes in general statuses [30, 32, 33, 43] used FIQ for the outcomes. The result of this review implies that acupuncture can be used to manage various accompanying symptoms rather than simply using it as a means to reduce pain in FMS.

### 4.2. Comparison with Other Systematic Reviews and Strength of This Systematic Review

As far as we know, in the previous systematic review by Deare et al. [29], the first meta-analysis on the effects of verum acupuncture and sham acupuncture in FMS indicated that the efficacy of acupuncture was negative for all symptoms. In their review, acupuncture was divided into two subgroups, i.e., manual acupuncture and electroacupuncture, and the effect sizes were calculated based on the final values of mainly VAS from the RCTs conducted prior to 2010. We, however, divided subgroups according to the type of sham acupuncture applied, and the effect sizes were estimated based on the changed values, which were measured by validated scales.

### 4.3. Limitations

There are some limitations in this systematic review and meta-analysis. Of the included studies, five RCTs [12, 21, 30, 41, 43] reported the results of the participants' blinding assessment. Given that practitioner blinding is impossible in the acupuncture studies, the credibility of the trials then should rely on participant and outcome assessment blinding. Despite the efforts to maintain appropriate blinding, a small number of participants can sense the group they were assigned to. Thus, future trials should take caution to make sure if the participant blinding was well implemented and maintained. Another limitation is that despite the fact that FMS is a chronic disease, we could not find a sufficient number of long-term follow-up studies for FMS, so we could only postulate the short-term effects.

## 5. Conclusions

We conclude that verum acupuncture compared with sham acupuncture has a short-term efficacy on reducing pain, as well as improving sleep quality and general status in FMS patients. However, evidence quality of moderate level in pain relief made our confidence in the effect estimate limited. On the other hand, we could not find any evidence that acupuncture has the ability to improve fatigue in FMS.

## Figures and Tables

**Table 1:** Summary of the included studies.

Study ID	Sample size		Intervention			Outcome	Participant's blinding assessment
	Acupuncture group (M : F)	Control group (M : F)	Duration	Acupuncture group	Control group		
*Included in meta-analysis*							
Deluze [42]	36 (3 : 33)	34 (13 : 21)	6 sessions over 3 weeks	*Electroacupuncture*: four common points (the first dorsal interosseous muscle of the hand and the anterior tibial muscle on both sides) + 6 other points (according to patient's symptoms and pattern)	*Nonacupoint electroacupuncture*: 20 mm away from the electroacupuncture points	*[Pain]*: VAS, pain threshold, regional pain score *[Sleep quality]*: VAS *[General status]*: patient's appreciation, evaluating physician's appreciation, *[Others]*: VAS of morning stiffness, number of analgesic tablets during the last week	Not reported
Harris et al. [21]	29 (0 : 29)	(1) 30 (3 : 27) (2) 28 (4 : 24) (3) 27 (1:26)	18 sessions over 9 weeks	*Manual acupuncture*: GV20, LI11, LI4, GB34, ST36, SP6, LR3, Shenmen manipulation for De Qi (−)	(1) *Simulated acupuncture*: same points as the verum acupuncture group, but the skin was not pierced. (2) *Nonacupoint acupuncture*: nonacupoints determined by acupuncturists. (3) *Simulated acupuncture on nonacupoint*: combination of 1 and 2	*[Pain]*: NRS *[Fatigue]*: MDFI *[Others]*: SF-36	The participants remained blinded at week 4 (Fisher's exact *χ*^2^ = 7.531, **P** = **0.259**)
Martin [43]	25 (0 : 25)	25 (1 : 24)	6 sessions over 2-3 weeks	*Electroacupuncture*: LI4, ST36, LR2, SP6, PC6, HT7, axial paramedian points along the bladder meridian at the cervical or lumbar spine. The sensation of De Qi or needle grab was not elicited	*Simulated electroacupuncture*: same points as the verum acupuncture group, but the skin was not pierced	*[Pain]*: pain of FIQ, pain of MPI *[Sleep quality]*: sleep of FIQ *[Fatigue]*: fatigue of FIQ *[General status]*: FIQ *[Others]*: physical function, well-being, stiffness and anxiety, depression of FIQ, interference, life control, affective distress, and general activity level of MPI	Only 7 were correct in the control group (28%)
Harris [41]	10 (0 : 10)	10 (0 : 10)	18 sessions over 9 weeks	*Manual acupuncture*: GV20, LI11, LI4, GB34, SP6, LR3, Shenmen, ST36 manipulation for De Qi (+)	*Unclassified acupuncture*: non-skin-penetrating pricking sensation within similar body locations as the TCM acupuncture points	*[Pain]*: SF-MPQ *[Others]*: positron emission tomography	Only 1 was correct in the control group
Stival et al. [31]	21 (4 : 17)	15 (1 : 14)	1 session	*Manual acupuncture*: LI4, ST36, LR2, SP6, PC6, HT7	*Nonacupoint acupuncture*: 15 mm left away from the TCM acupuncture point	*[Pain]*: VAS	Not reported
Vas et al. [30]	82 (0 : 82)	82 (0 : 82)	9 sessions over 9 weeks	*Manual acupuncture*: 8 common points (LI4, PC6, SP6, LR3, BL18, BL20, SP6, GB34) + other points (according to patient's symptoms and, pattern) manipulation for De Qi (+)	*Simulated acupuncture on inappropriate acupoints*: acupuncture simulation (through guide tube) on the dorsal and lumbar regions	*[Pain]*: VAS, pressure pain threshold, number of tender points *[General status]*: FIQ *[Others]*: depression of HAM, physical and mental of SF-12	Over 75% of the participants in both groups were confident that the intervention was real
Ugurlu et al. [33]	25 (0 : 25)	25 (0 : 25)	12 sessions over 8 weeks	*Manual acupuncture*: LI4, ST36, LR3, GB41, GB34, GB20, SI3, SI4, BL62, BL10, SP6, HT7, GV20, GV14, KI27, CV6, PC6 Manipulation for De Qi (−)	*Simulated acupuncture*: park sham devices	*[Pain]*: VAS *[fatigue]*: FSS *[general status]*: FIQ *[others]*: SF-36, BDI	Not reported
Karatay et al. [32]	25 (0 : 25)	(1) 25 (0 : 25) (2) 25 (0 : 25)	8 sessions over 4 weeks	*Manual acupuncture*: GV14, SI15, LI4, LI11, HT7, PC6, CV6, LR3, ST36, and SP6	(1) *Simulated acupuncture*: blocking by bandage at the same points as TCM acupuncture. (2) *Nonacupoint acupuncture*: 10–20 mm away from the TCM acupuncture points	*[Pain]*: VAS, number of tender points, pain of NHP *[Sleep quality]*: sleep of NHP *[General status]*: FIQ *[Others]*: SF-36, BDI, NHP, serotonin, substance P	Not reported

*Excluded from meta-analysis*							
Assefi et al. [12]	25 (3 : 22)	(1) 25 (1 : 24) (2) 24 (0 : 24) (3) 25 (1 : 24)	24 sessions over 12 weeks	*Manual acupuncture*: LI11, SP9, CV12, ST25, K7, TE5, Yintang, BL43, BL44, BL17, BL18, BL20, BL22, KI7	(1) *Simulated acupuncture*: toothpick in the guide tube at identical points as the verum acupuncture group (2) *Nonacupoint acupuncture*: body locations not recognized as true acupoints or meridians (3) *Acupuncture on points unrelated to FMS*: acupoints for irregular menses or “early menses due to blood heat”	*[Pain]*: VAS *[fatigue]*: VAS *[Sleep quality]*: VAS *[Others]*: well-being of VAS and physical and mental of SF-36	32% of participants believed they were receiving acupuncture specifically designed for fibromyalgia
Sprott [39]	10	(1) 10 (2) 10	6 sessions over 2 weeks	*Manual acupuncture*: various acupoints according to patient's symptoms and pattern	(1) *Simulated laser acupuncture*: disconnected laser equipment (2) *Waitlist*	*[Pain]*: number of positive tender points, VAS, pain threshold *[General status]*: interview	Not reported

VAS, visual analog scale; NRS, numerical rating scale; MDFI, multidimensional fatigue inventory; SF-36, 36-item short-form survey 36; FIQ, fibromyalgia impact questionnaire; MPI, multidisciplinary pain inventory; SF-MPQ, short form of the McGill pain questionnaire; HAM, Hamilton test score; SF-12, 12-item short-form survey; FSS, fatigue severity scale; BDI, Beck depression scale; HNP, Nottingham health profile; CPF, composite physical function scale.

**Table 2:** The quality of evidence.

Outcome	Certainty assessments						Summary of finding	
	Study design	Risk of bias	Inconsistency	Indirectness	Imprecision	Other considerations	Effect (95% CI)	Certainty
Intensity of pain	Verum acupuncture versus sham acupuncture							
	RCT	Not serious^a^	Serious^b^	Not serious^c^	Not serious^d^	None^e^	SMD −0.49 (−0.79∼−0.2)	⊕⊕⊕◯ MODERATE
	Verum acupuncture versus simulated acupuncture on appropriate points							
	RCT	Not serious^a^	Very serious^f^	Not serious^c^	Serious^g^	None^e^	SMD −0.54 (−1.19 ∼ 0.1)	⊕◯◯◯ VERY LOW
	Verum acupuncture versus acupuncture on inappropriate points							
	RCT	Not serious^a^	Serious^b^	Not serious^c^	Serious^h^	None^e^	SMD −0.63 (−1.23∼−0.04)	⊕⊕◯◯ LOW
	Verum acupuncture versus simulated acupuncture on inappropriate points							
	RCT	Not serious^a^	Not serious^i^	Not serious^c^	Not serious^d^	None^e^	SMD −0.37 (−0.67∼−0.08)	⊕⊕⊕⊕ HIGH

Fatigue	Verum acupuncture versus sham acupuncture							
	RCT	Serious^j^	Not serious^j^	Not serious^c^	Serious^k^	None^e^	SMD −0.1 (−0.51 ∼ 0.3)	⊕⊕◯◯ LOW

Sleep quality	Verum acupuncture versus sham acupuncture							
	RCT	Not serious^a^	Not serious^j^	Not serious^c^	Not serious^d^	None^e^	SMD −0.46 (−0.75∼−0.18)	⊕⊕⊕⊕ HIGH

General status	Verum acupuncture versus sham acupuncture							
	RCT	Not serious^a^	Not serious^j^	Not serious^c^	Not serious^d^	None^e^	SMD −0.69 (−0.91∼−0.47)	⊕⊕⊕⊕ HIGH
	Verum acupuncture versus simulated acupuncture on appropriate points							
	RCT	Not serious^a^	Not serious^j^	Not serious^c^	Not serious^d^	None^e^	SMD −0.71 (−1.04∼−0.39)	⊕⊕⊕⊕ HIGH
	Verum acupuncture versus acupuncture on inappropriate points							
	RCT	Not serious^a^	Not serious^j^	Not serious^c^	Serious^l^	None^e^	SMD −0.79 (−1.22∼−0.35)	⊕⊕⊕◯ MODERATE

RCT, randomized controlled trial; CI, confidence interval; SMD, standardized mean difference. ^a^The proportion of studies assessed as high risk of bias was less than 25%; ^b^*I*^2^ was 50% or more and less than 75%; ^c^directness was undoubted; ^d^total sample size was more than 200, and 95% CI of SMD did not cross zero; ^e^publication bias was not identified; ^f^*I*^2^ was 75% or more; ^g^total sample size was in the range of 101 to 200, and 95% CI of SMD crossed zero; ^h^total sample size was in the range of 101 to 200; ^i^*I*^2^ was less than 50%; ^j^the proportion of studies assessed as high risk of bias is in the range of 25% to 50%; ^k^total sample size was more than 200, and 95% CI of SMD crossed zero; ^l^total sample size was less than 100.

## Supplementary Materials

Supplementary Materials The keywords used to search each database are attached as the Appendix.
