# Correction to “Comparing Verum and Sham Acupuncture in Fibromyalgia Syndrome: A Systematic Review and Meta‐Analysis”

**DOI:** 10.1155/ecam/9896260

**Published:** 2026-08-09

J. Kim, S.‐R. Kim, H. Lee, and D.‐H. Nam, “Comparing Verum and Sham Acupuncture in Fibromyalgia Syndrome: A Systematic Review and Meta‐Analysis,” *Evidence-Based Complementary and Alternative Medicine* 2019, no. 1 (2019): 1–13, https://doi.org/10.1155/2019/8757685.

In the article titled “Comparing Verum and Sham Acupuncture in Fibromyalgia Syndrome: A Systematic Review and Meta‐Analysis”, there was an error in section 3.3 Primary Outcome (Intensity of Pain).

This error is shown below:

‘When the analyses were restricted to the RCTs comparing verum acupuncture with SA‐AP [32, 33, 41, 43] and A‐IP [31, 32, 41, 42], separately, the beneficial pain relief effect remained (SA‐AP: SMD −0.54 (95% CI: −0.19, 0.10), *Z* = 1.64, *P* = 0.10; A‐IP: SMD −0.63 (95% CI: −1.23, −0.04), *Z* = 2.07, *P* = 0.04), but the heterogeneities were all substantial (SA‐AP: *I* ^2^ = 75%; A‐IP: *I* ^2^ = 68%).’

The statement should read:

‘When the analyses were restricted to the RCTs comparing verum acupuncture with SA‐AP [32, 33, 41, 43] and A‐IP [31, 32, 41, 42], separately, the pain relief effect was not statistically significant for the SA‐AP comparison (SMD −0.54 (95% CI: ‐1.19, 0.10), *Z* = 1.64, *P* = 0.10) but was significant for the A‐IP comparison (SMD −0.63 (95% CI: −1.23, −0.04), *Z* = 2.07, *P* = 0.04). However, the heterogeneity was substantial in both subgroups (SA‐AP: *I* ^2^ = 75%; A‐IP: *I* ^2^ = 68%).’

We apologize for this error.

