# Supplementary material for: Comparing Verum and Sham Acupuncture in Fibromyalgia Syndrome: A Systematic Review and Meta-Analysis
Source: Evid Based Complement Alternat Med. 2019 Aug 25;2019:8757685. doi: 10.1155/2019/8757685 (PMC6732586; doi:10.1155/2019/8757685)
Supplement: Supplementary Materials — The keywords used to search each database are attached as the Appendix. [file 8757685.f1.docx]

Appendix 1. Searching keywords for each database

| Database | Keywords |
| --- | --- |
| PubMed | ("fibromyalgia" [MESH] OR fibromyositis OR "fibromyalgia-fibromyositis syndrome" OR "myofascial pain syndrome") and (acupuncture OR auriculotherapy OR electroacupuncture OR acupoint OR needling) |
| Cochrane Central | ("fibromyalgia" [MESH] OR fibromyositis OR "fibromyalgia-fibromyositis syndrome" OR "myofascial pain syndrome") and (acupuncture OR auriculotherapy OR electro-acupuncture OR electroacupuncture OR acupoint OR needling), in Trials (Word variations have been searched) |
| Embase | ('fibromyalgia'/exp OR fibromyositis OR 'fibromyalgia-fibromyositis syndrome' OR 'myofascial pain syndrome') AND ('acupuncture'/exp OR 'acupuncture' OR 'acupuncture therapy' OR 'auriculotherapy' OR 'point, acupuncture' OR auriculoacupucture OR electroacupuncture OR acupoint OR 'needling'/exp) |
| PsycINFO | (fibromyalgia OR fibromyositis OR (fibromyalgia-fibromyositis syndrome) OR (myofascial pain syndrome)) AND (acupuncture OR auriculotherapy OR electroacupuncture OR electro-acupuncture OR acupoint OR needling) |
| CNKI | SU=(fibromyalgia + fibrositis + fibromyositis + myofascial pain syndrome + myofascial trigger point) and SU=(acupuncture + moxibustion + auricular acupoint + auriculotherapy + electro-acupuncture) |
| VIP | M=(fibromyalgia OR fibrositis OR fibromyositis OR myofascial pain syndrome OR myofascial trigger point) AND M=(acupuncture OR moxibustion OR auricular acupoint OR auriculotherapy OR electro-acupuncture) |
| OASIS | (fibromyalgia OR myofascial pain syndrome OR myofascial pain) AND acupuncture |
| KoreaMed | ( fibromyalgia [ALL] OR fibromyositis [ALL] ) AND ( acupuncture [ALL] OR auriculotherapy [ALL] OR electroacupuncture [ALL] OR acupoint [ALL] OR needle [ALL] ) |
| RISS | (fibromyalgia OR myofascial pain syndrome OR myofascial pain) AND acupuncture |
